# Supplementary material for: Polyketide synthase gene clusters in the ericoid mycorrhizal fungus Oidiodendron maius: genome-wide prediction, expression profiling, and functional characterization of knockout mutants
Source: Front Plant Sci. 2026 Sep 16;17:1924430. doi: 10.3389/fpls.2026.1924430 (PMC13623903; doi:10.3389/fpls.2026.1924430)
Supplement: Supplementary file 1 [file SupplementaryFile1.zip › Supplementary Materials/Supplementary Folder 1.zip!/Oidma1_AssemblyScaffolds_Repeatmasked/knownclusterblast/cluster55/jgi.p_Oidma1_173688_mibig_hits.html]

| MIBiG Protein | Description | MIBiG Cluster | MiBiG Product | % ID | % Coverage | BLAST Score | E-value |
| --- | --- | --- | --- | --- | --- | --- | --- |
| EAL89343.1 | toxin\_biosynthesis\_protein,\_putative | BGC0001403 | Trypacidin\_biosynthetic\_gene\_cluster | 64 | 98.4905660377 | 333 | 2.5e-91 |
| AGO59035.1 | PtaF | BGC0000121 | Pestheic\_acid\_biosynthetic\_gene\_cluster | 58 | 98.8679245283 | 311 | 1.3e-84 |
| CBF90090.1 | toxin\_biosynthesis\_protein,\_putative\_(AFU\_orthologue;\_AFUA\_4G14520) | BGC0001271 | Emericellin\_biosynthetic\_gene\_cluster | 51 | 99.2452830189 | 264 | 1.9e-70 |
| AAS90016.1 | OrdB | BGC0000007 | Aflatoxin\_biosynthetic\_gene\_cluster | 40 | 96.6037735849 | 202 | 6.7e-52 |
| AAS90084.1 | OrdB | BGC0000010 | Aflatoxin\_biosynthetic\_gene\_cluster | 39 | 96.6037735849 | 200 | 2.6e-51 |
| AAS90038.1 | OrdB | BGC0000008 | Aflatoxin\_biosynthetic\_gene\_cluster | 39 | 96.6037735849 | 198 | 1.3e-50 |
| BAE71334.1 | oxidreductase\_B/\_cytochrome\_P450\_monooxygenase | BGC0000004 | Aflatoxin\_biosynthetic\_gene\_cluster | 39 | 96.6037735849 | 197 | 2.2e-50 |
| ACH72895.1 | AflX | BGC0000011 | Aflatoxin/sterigmatocystin\_biosynthetic\_gene\_cluster | 40 | 96.2264150943 | 196 | 3.7e-50 |
| AAS90109.1 | OrdB | BGC0000006 | Aflatoxin\_biosynthetic\_gene\_cluster | 39 | 96.6037735849 | 196 | 4.8e-50 |
| AAS90064.1 | OrdB | BGC0000009 | Aflatoxin\_biosynthetic\_gene\_cluster | 38 | 96.6037735849 | 193 | 4.1e-49 |
| AAC49202.1 |  | BGC0000152 | Sterigmatocystin\_biosynthetic\_gene\_cluster | 40 | 98.1132075472 | 190 | 2e-48 |
